# Centering equity and lived experience: implementing a community-based research grant on cannabis and mental health

**DOI:** 10.1186/s12939-022-01722-4

**Published:** 2022-08-20

**Authors:** Pamela Obegu, Julia Armstrong, Mary Bartram

**Affiliations:** 1 School of Public Health and Epidemiology, University of Ottawa, Ottawa, Ontario Canada; 2 Mental Health Commission of Canada, Ottawa, Ontario Canada

**Keywords:** Health equity, Community-based research, Mental health research, Research investment, Lived experience, Cannabis, Research productivity, Priority populations, Capacity bridging

## Abstract

**Background:**

Mental health research in Canada is not only underfunded but there remains an inequitable distribution of funding to address unmet needs especially in clinical and applied research. In 2018, the legalization of cannabis for non-medical use in Canada sparked the need to examine the relationship between cannabis use and mental health. The federal government allocated $10 M over 5 years to the Mental Health Commission of Canada (MHCC), a pan-Canadian health organization funded at arm’s length by the federal government.

**Methods:**

In 2020, the MHCC implemented an innovative community-based research (CBR) program to investigate this relationship among priority populations including people who use cannabis and live with mental illness, First Nations, Inuit and Métis, two-spirit, lesbian, gay, bisexual, trans and/or queer (2SLGBTQ+) individuals, and racialized populations. Extensive consultations, a scoping review and an environmental scan set the research agenda. Key program components included a review committee with representation from diverse priority populations, extensive proposal-writing support for applicants, and capacity bridging workshops for the 14 funded projects.

**Results:**

Of the 14 funded research projects, 6 focus on and are led by Indigenous communities, 5 focus on other equity-seeking populations, and 9 explore the perceived patterns, influence and effects of use including benefits and harms. Lessons learned include the importance of a health equity lens and diverse sources of knowledge setting the CBR research agenda. In addition to capacity bridging that promote equitable roles among knowledge co-producers as well as the critical role of organizational support in increasing research productivity, especially in the area of mental health and cannabis use where there is a need for more applied research.

**Conclusion:**

Centering equity and lived and living experience strengthened the rationale for investments and ensured user-led evidence generation and utilization – a key public health gain. Organizational support for proposal development and capacity bridging yields significant value that can be replicated in future CBR initiatives.

## Introduction

The global underinvestment in mental health research is well-known, with several calls for action from both low and high income countries for increased funding and mental health research prioritization [1–4]. In Canada, mental health research grants are about ten times smaller when compared to the United States and the United Kingdom, and the majority of mental health research investments focus on hospital-related service provision rather than applied research with a focus on prevention [3]. In this research environment, community-based research (CBR) that is led by equity-seeking populations and centred in lived and living experience is often found lower down the list of mental health research priorities. As recommended in the Mental Health Strategy for Canada [5], meaningful engagement of people with lived and living experience (PWLLE) in policy research and planning is critical for advancing system transformation, and for ensuring that reforms address high levels of unmet mental health needs [6, 7].

Following the legalization of cannabis for non-medical use in Canada in 2018, the Mental Health Commission of Canada (MHCC), a pan-Canadian health organization funded by but at arms length from the federal government, received $10 million from the federal government to study the impact of cannabis on the mental health of Canadians [8]. The MHCC first commissioned an environmental scan and scoping review conducted by the University of Calgary to get the full picture of cannabis use and mental health outcomes in the Canadian context [9]. Findings from the scan and review identified the following research gaps and priorities: (i) longitudinal studies to help clarify the complex relationship between cannabis use and mental health outcomes; (ii) studies that explore the different modes of consumption of cannabis and account for such differences; (iii) a focus on specific populations as very little research focused on outcomes in specific groups such as seniors, two-Spirit, lesbian, gay, bisexual, trans and/or queer (2SLGBTQ+) individuals, Indigenous communities, and immigrant, refugee, ethnocultural, and racialized populations – with considerations of social determinants of health, including age, sex and gender, income, education level, and past experiences of trauma; and (iv) qualitative studies that centre the insights from PWLLE of cannabis use, including research into the potential benefits of casual and moderate cannabis use. These recommendations shaped two streams of research grants: academic research awarded through a traditional peer review process facilitated by the Canadian Institutes for Health Research, and a CBR initiative overseen directly by the MHCC.

To identify further priorities for the CBR program on cannabis and mental health, the MHCC held two community forums in 2019. One was held in Ottawa with a highly diverse group of experts, including PWLLE of substance use and/or mental health problems and illnesses, service providers, family members, community-based researchers, and policy makers [10]. The other was focused on cannabis and mental health research priorities for Inuit and was held in Happy Valley-Goose Bay in Labrador [11]. Participants of the fora validated the recommendations of the environmental scan and scoping review and further recommended that CBR be strength-based, co-produced rather than extractive, grounded in local culture and language, culturally safe, and sustainable. This paper seeks to describe the implementation of an innovative CBR research grant program among priority populations who use cannabis and/or live with mental illness in Canada, as well as the lessons learned in centering equity and lived experience.

## Approach

Effective and meaningful engagement is at the core of CBR and is a guiding principle for academic and community researchers who co-produce knowledge with community stakeholders [12]. Based on the identified research priorities and principles, the MHCC’s CBR program was initiated in 2020 to address knowledge gaps in the relationship between cannabis and mental health and to build research capacity among identified priority populations at the community level. Key program components included a request for proposals (RFP) that centred equity and lived and living experience [13], extensive proposal-writing support for applicants from a network of CBR hubs, ample submission time (including an extension due to COVID-19), a review committee with representation from diverse priority populations, and capacity bridging workshops for the 14 funded projects.

The RFP built on the recommendations from the scan and community fora and was further refined through consultation with diverse stakeholders and experts in CBR and equitable research granting processes. For example, the research priorities on cannabis and mental health named in the RFP emphasized factors influencing cannabis use without pre-judging whether such factors were beneficial or harmful, and specifically encouraged applications on the impact of criminalization – drawing from the recommendations from the scan and community fora. Principles for CBR were clearly identified and emphasized, including a requirement that all projects be grounded in local culture and that research teams demonstrate equitable decision making and roles. The RFP was promoted through the MHCC’s networks (including participants of the community fora) and a webinar [14].

Soon after the RFP was released, a public webinar was held in March 2020 that introduced several CBR research hubs that the MHCC engaged to support proposal development among interested applicants with varying levels of research capacity. These hubs included the Wellesley Institute (Greater Toronto Area), the Centre for Community Based Research (University of Waterloo), Inuit Tapiriit Kanatami (the national representational organization for Inuit in Canada), the National Collaborating Centre for Determinants of Health (Saint Francis Xavier University), the National Collaborating Centre for Indigenous Health (University of Northern British Columbia), and the Centre for Healthy Communities (University of Alberta). Teams were invited to request proposal development support from the MHCC, who matched them to the hub that best aligned with their needs. The MHCC did not provide any direct proposal development support so as to remain impartial when making funding decisions.

At the time of the original launch of the RFP in late February 2020, communities had 8 weeks to engage in the proposal development process. The onset of the COVID-19 pandemic in mid-March had a significant impact on communities everywhere. After first consulting with community representatives regarding whether to postpone the CBR call altogether, the MHCC decided to proceed but extended the deadline by 5 weeks (13 weeks total) to give communities additional time to develop their proposals. By the close of the RFP at the end of May 2020, the MHCC received 60 proposals from across the country.

To further centre equity and lived experience, the MHCC recruited a team of 12 independent reviewers comprised of members of each of the priority populations identified in the RFP, community-based and academic researchers, and PWLLE. Each proposal was assigned to 2 reviewers. Final recommendations were made following deliberations with the full team of reviewers. While the original call was for 12 funded projects, the MHCC was able to reallocate funds to support 14 CBR projects on cannabis and mental health led by priority populations at the community level. All projects set out to explore perceived patterns, influence and effects of use including benefits and harms among Indigenous peoples, two spirit, lesbian, gay, bisexual, trans and/or queer (2SLGBTQ+) individuals, racialized populations, and communities experiencing multiple layers of oppression. Six projects were Indigenous-led and sought to explore the impact of cannabis use on Indigenous communities (including Indigenous elders and youth) and to develop harm reduction strategies through Indigenous ways of knowing.

Funded research teams were supported through a monthly series of virtual training workshops facilitated by the Centre for Community Based Research over a 5-month period (between November 2020 and March 2021). During the CBR training workshops, a community of practice approach was adopted to bridge the capacity of workshop participants that comprised of early-stage and seasoned academics, community-based researchers, PWLLE, and priority populations, with the aim to continuously learn from and share with one another during the training and beyond. The strategy of prolonged engagement as outlined by Glesne & Peshkin [15] was used to strengthen and sustain capacity, and to give participants adequate time to express their views.

## Lessons learned

Despite the challenges of the COVID-19 pandemic, all 14 CBR projects initiated their studies and are on track to address significant gaps in knowledge regarding the relationship between cannabis and mental health among priority populations. While it is too soon to evaluate the impact of the research findings, 4 interconnecting lessons learned regarding the implementation of the CBR granting process are explored below.

First, a health equity lens and diverse sources of knowledge were critical for setting the CBR research agenda. These sources of knowledge include a review of existing evidence that specifically analyzed gaps in research among priority populations, complemented by broad engagement with PWLLE of inequity and of the complex relationship between cannabis and mental health. This rigorous process not only ensured that the research will be relevant to communities but also reinforced the case for investment in CBR on par with more traditional academic research.

Second, the value of organizational support for community-based researchers cannot be overemphasized as it plays a critical role in increasing research productivity, especially in the area of mental health and cannabis use where there is a need for more applied research [3, 16]. The MHCC’s support for proposal development, facilitated through the 6 CBR hubs, led to the high response of 60 proposals received from communities and priority populations. This response was especially significant as it occurred during the first wave of the COVID-19 pandemic. For the 14 funded CBR teams, the monthly virtual training workshops also played a critical role in supporting the teams to get off to a strong start. These experiences are consistent with the documented role and benefit of organizational support in ensuring increased involvement in research production and utilization of research evidence among key players in public health environments [17, 18]. Those who feel that they have the expectation and organizational support to do research have been found to be more productive than those who do not have the benefit of this support [19]. When there is a will by organizations to support CBR through strong community partnerships that allow for active community participation, often-overlooked populations are able to embrace research opportunities.

Third, the involvement of PWLLE throughout the research granting process significantly strengthened the relevance and credibility of the initiative overall. Typically, health research funding programs and decisions are ‘academic’ in nature and in scope [20]. As a result, PWLLE rarely make it through these competitions to receive funding to undertake applied mental health research investigations – an area that remains understudied. PWLLE who were engaged to shape the research agenda through the community fora and the peer reviewers who identified as PWLLE pointed to the value of representation of communities under study during the program planning and proposal review process – indicating that such representation has enhanced the quality and relevance of the funded research projects. This engagement will ultimately enrich the quality of evidence generated regarding the relationship between cannabis and mental health. Involvement of PWLLE throughout the consultation, planning, and implementation stages further increased the readiness of PWLLE and priority populations to mobilize the CBR findings. The MHCC’s CBR granting process exemplifies an avenue for co-produced and user-led research.  Of what purpose is research if it is not utilized?

Lastly, capacity bridging can promote equitable roles among academic and community researchers who co-produce knowledge with community stakeholders [21]. The monthly series of virtual training workshops for the 14 funded research teams took on a community of practice approach to bridge the research capacity of workshop participants that comprised of early-stage and seasoned academics, community-based researchers, PWLLE and members of priority populations. These workshops were well received by participants and fostered a better understanding of CBR fundamentals and stronger relationships within and across teams. Participants also learned from each other about the importance of tailoring community engagement to the unique cultural practices of their communities including Indigenous ways of knowing, rather than imposing a standard approach.

## Conclusion

The MHCC’s CBR granting program on cannabis and mental health was greatly enriched through the centering of equity and lived and living experience. Not only did this program address inequities in mental health research – it was implemented with success during a pandemic. Valuable lessons were gained about how to implement innovative research by drawing on key program components including using diverse sources of evidence to set the research priorities, the significance of a review committee with representation from diverse priority populations, extensive proposal-writing support and time for applicants, and capacity bridging workshops for funded projects. Centering equity and lived experience strengthens the rationale for investments and ensures user-led evidence generation and utilization – a key public health gain. Organizational support for proposal development and capacity bridging yield significant value that can be replicated among policy practitioners in mental health and substance use research and service provision.

## Data Availability

Not applicable.

## References

[1] Patel V. Mental health research funding: too little, too inequitable, too skewed. Lancet Psychiatry. Retrieved from. 2020. 10.1016/S2215-0366(20)30471-5. 10.1016/S2215-0366(20)30471-5 33242399

[2] Tomlinson M, Rudan I, Saxena S, Swartz L, Tsai AC, Patel V (2009). Setting priorities for global mental health research. Bull World Health Organ.

[3] Woelbert E, White R, Lundell-Smith K, Grant J, Kemmer D (2020). The inequities of mental Health Research (IAMHRF). Digital Science.

[4] World Health Organization (2013). Investing in mental health: evidence for action.

[5] Mental Health Commission of Canada (2012). The Mental Health Strategy for Canada.

[6] Jacquez F, Vaughn LM, Wagner E (2013). Youth as partners, participants or passive recipients: A review of children and adolescents in community-based participatory research (CBPR). Am J Community Psychol.

[7] MacLean S, MacKie C, Hatcher S (2018). Involving people with lived experience in research on suicide prevention. CMAJ.

[8] Mental Health Commission of Canada (2018). MHCC receives $10 million to study cannabis use.

[9] Mental Health Commission of Canada (2019). Cannabis and Mental Health: Priorities for research in Canada.

[10] Mental Health Commission of Canada (2019). Shaping Future Investments in Community-Based Research on Cannabis and Mental Health.

[11] Mental Health Commission of Canada (2019). Inuit Forum on Cannabis and Mental Health.

[12] Ochocka J, Janzen R, Macaulay A, Hawkins L, De Grosbois S, Lydon M. Seeking meaningful engagement of community partners: Clarifying guiding principles of community-based research practice. In: CU Expo 2013. Corner Brook; 2013.

[13] Mental Health Commission of Canada (2020). Request for Proposals: Community-Based Research Projects in Cannabis and Mental Health.

[14] Mental Health Commission of Canada (2020). Community-Based Research Request for Proposals Information Session.

[15] Glesne C, Peshkin A (1992). Becoming qualitative researchers: an introduction.

[16] Nicholson K, Ganann R, Bookey-Bassett S, Baird LG, Garnett A, Marshall Z, Khan AI, Pirrie M, Sasseville M, Charif AB, Poitras M, Kyoon-Achan G, Dionne E, Hassani K, Stewart M (2020). Capacity building and mentorship among pan-Canadian early career researchers in community-based primary health care. Primary health care research & development.

[17] Mazzucca S, Parks RG, Tabak RG, Allen P, Dobbins M, Stamatakis KA, Brownson RC (2019). Research full report: assessing organizational supports for evidence-based decision making in local public health departments in the United States: development and psychometric properties of a new measure. J Public Health Manag Pract.

[18] Zardo P, Collie A (2014). Predicting research use in a public health policy environment: results of a logistic regression analysis. Implement Sci.

[19] Hoffmann K, Berg S, Koufogiannakis D (2017). Understanding factors that encourage research productivity in academic librarians. Evid Based Libr Inf Pract.

[20] Wang J, Veugelers R, Stephan P (2017). Bias against novelty in science: A cautionary tale for users of bibliometric indicators. Res Policy.

[21] Duddy J (2017). Capacity bridging: reciprocity at work in research.

